# Sarcomatoid carcinoma transformation in oral undifferentiated carcinoma following sequential immune combined targeted therapy: a case report

**DOI:** 10.3389/fimmu.2024.1484915

**Published:** 2024-10-24

**Authors:** Jieying Li, Xiaohong Zhan, Wei Shang, Kai Song

**Affiliations:** ^1^ Department of Oral and Maxillofacial Surgery, The Affiliated Hospital of Qingdao University, Qingdao, Shandong, China; ^2^ School of Stomatology, Qingdao University, Qingdao, Shandong, China; ^3^ Department of Pathology, The Affiliated Hospital of Qingdao University, Qingdao, Shandong, China

**Keywords:** undifferentiated carcinoma, sarcomatoid carcinoma, pathological transformation, immunotherapy, targeted therapy, conversion therapy

## Abstract

The diagnosis and treatment of head and neck undifferentiated carcinoma (HNUC) present significant challenges. Herein, we present the case of a patient with advanced HNUC who underwent conversion surgery following treatment with a combination of pembrolizumab and nimotuzumab. During therapy, histological transformation from undifferentiated to sarcomatoid carcinoma was detected at the primary site. This case not only highlights the potential of immune combination-targeted therapy to reduce tumour burden and increase the surgical options for patients, but also reveals the complex alterations in tumour biology that may occur during treatment. It emphasizes the necessity for routine pathological assessments throughout the therapeutic regimen to guide personalised therapeutic strategies and optimise patient prognoses.

## Introduction

Head and neck undifferentiated carcinoma (HNUC) is a rare subtype of head and neck cancer (HNC) characterised by low incidence and poor prognosis (1, 2). While HNUC is more common in the nasopharynx, its occurrence in the oral cavity is extremely rare (2–4). Despite exploration of multimodal therapies for HNUC, no consensus has been reached regarding the optimal treatment regimen or sequence. The 2022 first edition of the National Comprehensive Cancer Network (NCCN) guidelines recommends immune combination targeted therapy for special cases of head and neck squamous cell carcinoma (HNSCC). In some cases, Pembrolizumab (PD-1 inhibitor), is reported to have potential efficiency in treatment of HNUC (5). Although Sakamoto et al. reported a successful case of combined targeted therapy and chemotherapy for undifferentiated carcinoma (UC) of the tongue (6), the efficacy of combined immune and targeted therapies for UC of the oral cavity remains unclear.

Pathological transformation has been reported in various solid tumours and may be correlated with a poor prognosis (7, 8). Recently, immune and/or targeted therapy-induced pathological transformations have been associated with potential acquired resistance in patients (9). For example, the transformation of non-small cell lung cancer (NSCLC) to small-cell lung cancer (SCLC) represents a significant mechanism of resistance to chemotherapy, immunotherapy, and targeted therapy (10). Combined immune target-related pathological transformations are rare in HNC. Here, we report a rare case of a patient with advanced PD-L1+ and EGFR+ HNUC who underwent pathological transformation from HNUC to sarcomatoid carcinoma (SC) following combined immune and targeted treatment. This case evaluates the potential efficacy of combined immune and targeted therapy for UC of the oral cavity and subsequent treatment strategies following pathological transformation.

## Case report

**Figure 1:**
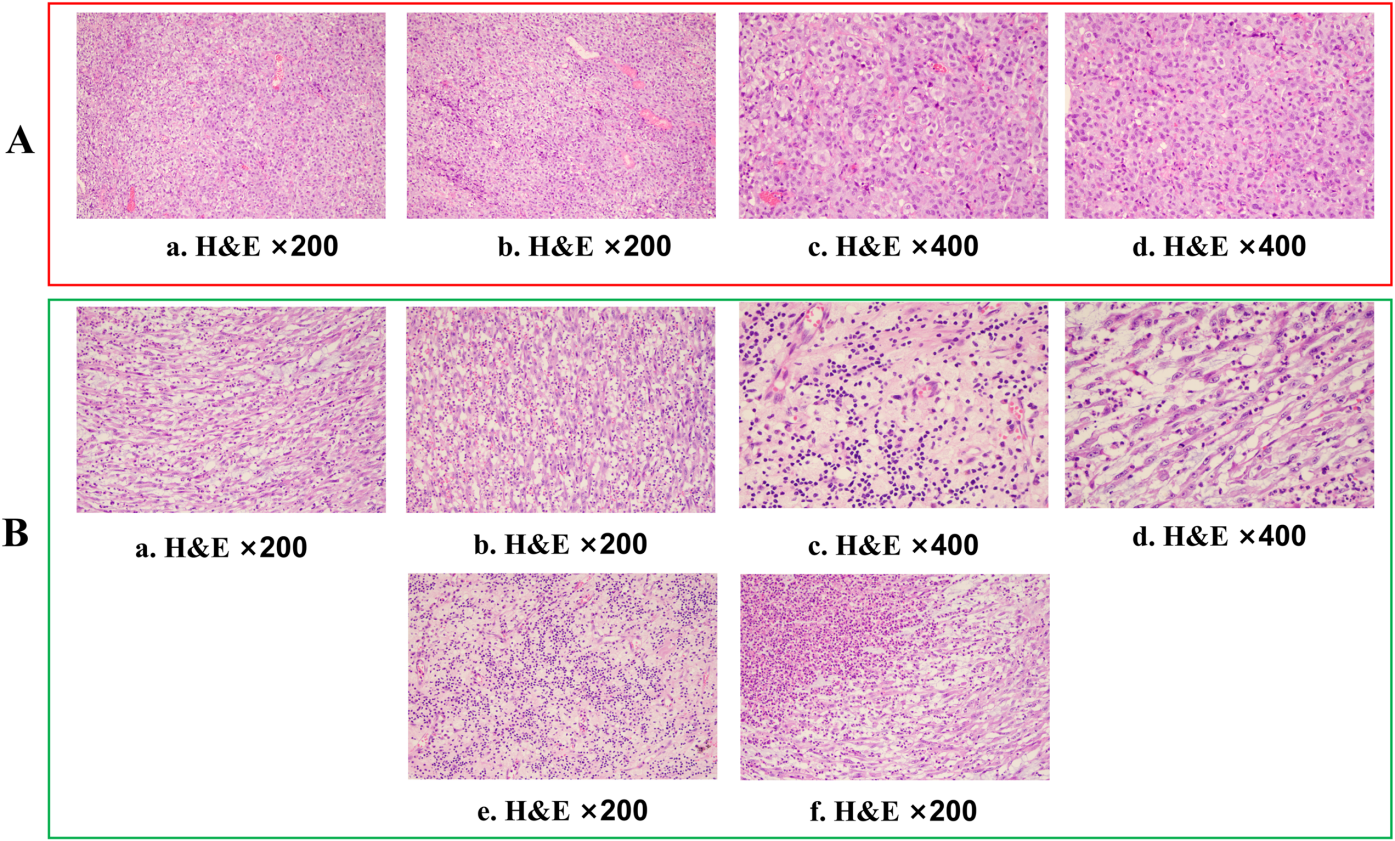
Histopathological assessment of tumour tissue. **(A)** Haematoxylin and eosin (H&E) staining (×200 and ×400) of a gingiva biopsy showed tumour cells with an epithelial-like morphology, diffusely distributed in sheets, with no evidence of squamous differentiation or glandular structures. (**B**, a–d). Haematoxylin and eosin (H&E) staining (×200 and ×400) of the gingival biopsy revealed that the tumour cells predominantly exhibited a spindle-shaped morphology. (e, f) In some areas, the tumour exhibited significant degeneration and necrosis, accompanied by histiocytic response and marked lymphocytic infiltration.

**Figure 2:**
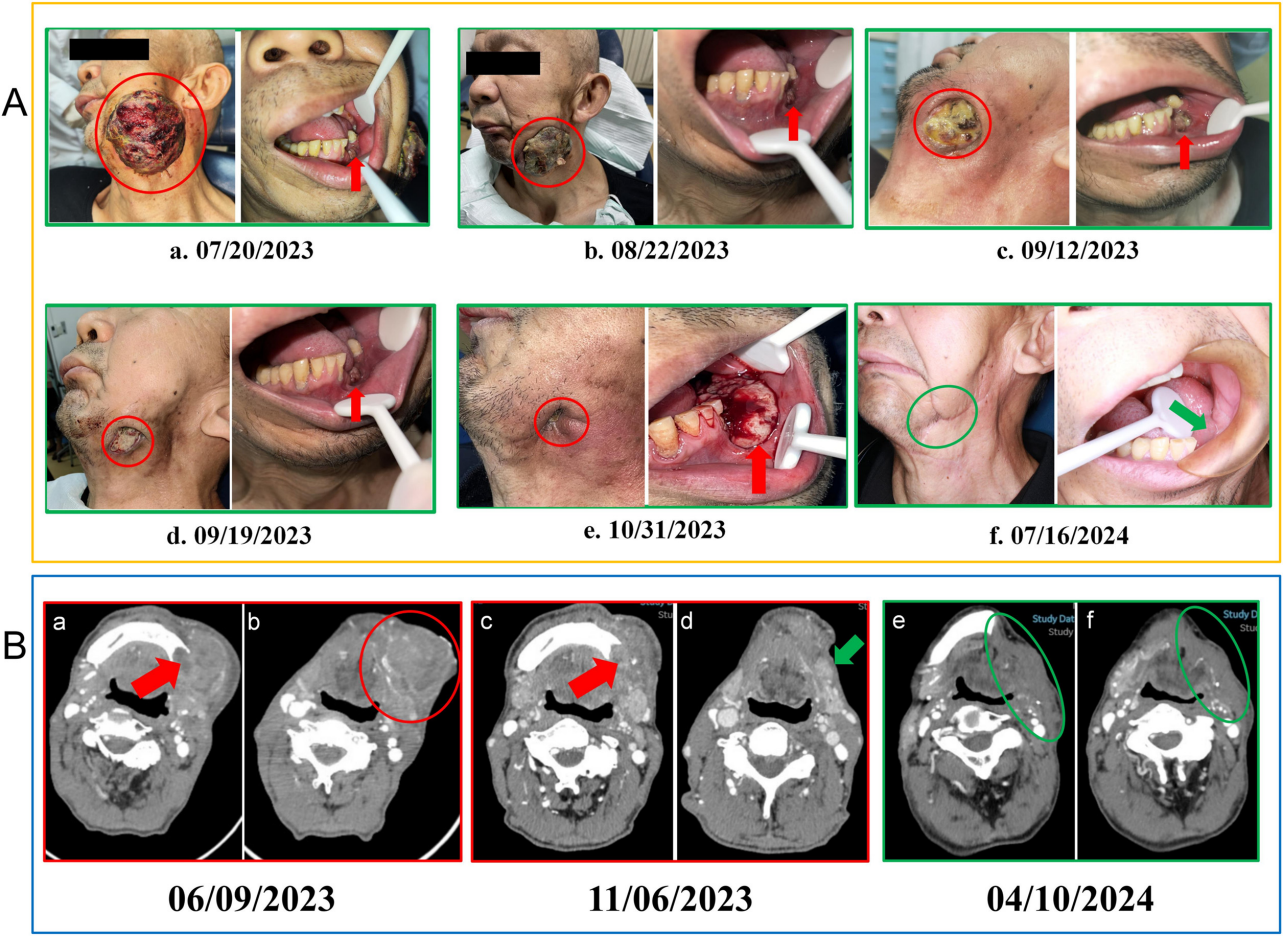
**(A)** Changes in the lesions during treatment. (a) Prior to treatment, the intraoral lesion displayed tooth loss in the left lower molar region with destruction of the alveolar ridge, while the left submandibular metastatic lesion showed surface ulceration and bleeding. (b) After two cycles of treatment, the intraoral lesion showed minor reduction, and the left submandibular metastatic lesion decreased significantly. (c) Nine days after three cycles, the intraoral lesion remained relatively unchanged, while the submandibular lesion demonstrated showed further reduction. (d) Sixteen days after three cycles, the intraoral lesion remained stable, and the submandibular lesion maintained a PR status. (e) Fourteen days after five cycles of treatment, the tumour in the left lower gingiva showed rapid growth, while the submandibular lesion had nearly disappeared. (f) Five months post-surgery, no recurrence of the lesion was observed. Red circles mark the extraoral lesion, red arrows point to the intraoral lesion, while green arrows and circles indicate the intraoral and extraoral wound status 5 months post-surgery. PR, partial response; CT, computed tomography; **(B)** Patient imaging examinations during the course of the disease. (a, b) At the time of initial diagnosis, a CT scan of the neck revealed osteolytic destruction of the left mandibular body and ramus, along with left-sided lymphadenopathy. (c, d) Following five cycles of combined immune-targeted therapy, no significant change was noted in the bony destruction, but the surrounding soft tissue mass had reduced. (e, f) A follow-up CT scan on April 10, 2023, five months post-surgery, showed no signs of recurrence. Red arrows indicate the mandibular destruction area, red circles mark the submandibular metastatic lesion, green arrows show the submandibular lesion after 5 cycles of combined targeted therapy, and green circles represent the original lesion site 5 months post-surgery.

A 60-year-old male patient, with a long history of smoking and alcohol consumption presented with a painful lesion on the left lower gingiva lasting over one year, and a left submandibular mass for more than one month. On May 31, 2023, he consulted the Department of Oral and Maxillofacial Surgery at the Affiliated Hospital of Qingdao University, where a pathological biopsy revealed a malignant tumour of the left lower gingiva (**Figures 1A**, a–d). An enhanced computed tomography (CT) scan of the neck showed osteolytic destruction in the body and ramus of the left mandible, measuring approximately 34mm x 42mm x 77mm, and multiple enlarged lymph nodes in the neck (**Figure 2B**, a-b). The patient initially declined the recommended surgical intervention and opted for chemotherapy instead. On June 10, 2023, he started chemotherapy with the TPF regimen (Docetaxel, 350mg, d1; Cisplatin, 60mg, d1-d2; Capecitabine, 1.5g, d1-d14) in the Oncology Department. However, on the sixth day of taking Capecitabine, the patient experienced seizures and developed severe anemia, requiring blood transfusions due to unstable vital signs. And Capecitabine was discontinued on June 16, 2023. Subsequently, chemotherapy was discontinued, and the patient chose to receive palliative care at home. The tumour size increased drastically, with the left submandibular mass growing rapidly. It eventually breached the skin, leading to ulceration and bleeding (**Figures 2A**, a).

**Figure 3:**
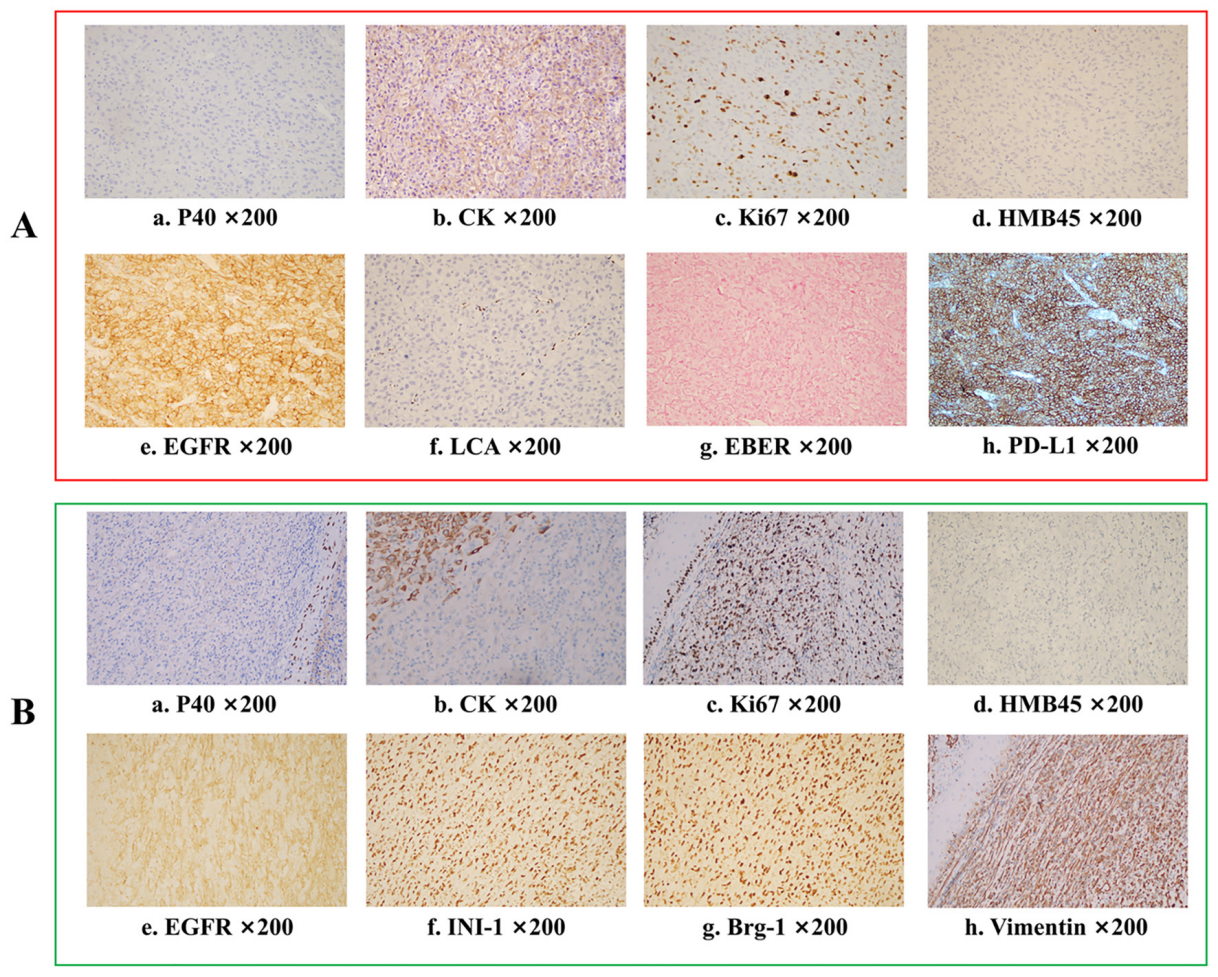
Immunohistochemistry analysis for tumours before and after pembrolizumab combined with nimotuzumab treatment. **(A)** Pathological results before treatment indicate undifferentiated carcinoma Ki-67 (+, 30%), CK (cytoplasmic +), CK5/6 (-), P40 (-), EBER (-), P16(-), HMB45(-), LCA(-), EMA(-), ERG(-), U145 PU.1(-), PD-L1(22C3)(CPS:80), EGFR (+). **(B)** Final pathological results after surgery showed a sarcomatoid carcinoma Ki-67 (+, 70%), CK (focal +), CK5/6 (-), p40 (-), p16 (-), HMB45 (-), p63 (-), S100(-), INI-1 (+), MyoD1 (-), Vimentin (+), Brg-1(+), p53 (++, 60%), EGFR (+).

On July 18, 2023, the patient presented to the oral emergency department with uncontrolled bleeding from the lesion. Hemostasis was achieved through packing and application of pressure. Microscopic examination of the biopsy tissue showed tumour cells with an epithelial-like morphology, diffusely distributed in sheets, with no evidence of squamous differentiation or glandular structures. Additional immunohistochemistry (IHC) on the biopsy sample of the patient’s intraoral lesion showed Ki-67 (+, 30%), CK (cytoplasmic +), CK5/6 (-), P40 (-), EBER (-), P16 (-), HMB45 (-), LCA (-), EMA (-), ERG (-), U145 PU.1 (-), PD-L1 (22C3) (CPS:80), and EGFR (+) (**Figures 3A**, a–h). Based on the histological morphology and IHC results, malignancies such as melanoma, lymphoma, and angiosarcoma were ruled out, and the patient was diagnosed with advanced-stage HNUC. Given the patient’s current overall condition, treatment tolerance, tumour imaging, and personal preference, a multidisciplinary team (MDT) recommended immune combined targeted therapy (pembrolizumab 200 mg, q3w, IV drip; nimotuzumab 400 mg, q3w, IV drip). The patient first received Pembrolizumab + Nivolumab on July 21, 2023, followed by subsequent cycles every 21 days: Second cycle on August 11, 2023, Third cycle on September 3, 2023, Fourth cycle on September 24, 2023, Fifth cycle on October 16, 2023.

**Figure 4:**
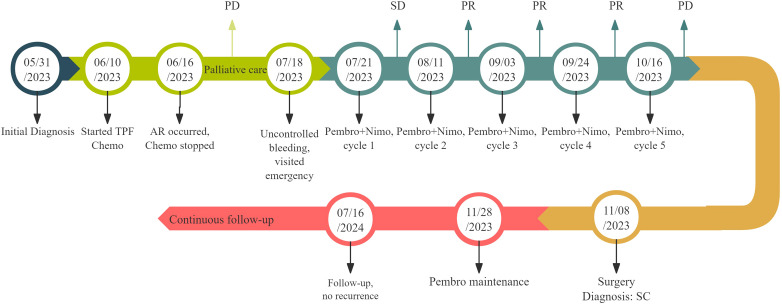
Timeline of clinical management and disease evolution. The patient began TPF Chemo on June 10, 2023. On June 16, 2023, severe AR (severe anemia) occurred, leading to the cessation of Chemo and the decision to opt for palliative care. During palliative treatment, the lesion gradually enlarged, and on July 18, 2023, the patient presented to the emergency department due to uncontrolled bleeding, which was managed with pressure bandaging. On July 21, 2023, the first cycle of Pembro combined with Nimo therapy was initiated, followed by the second cycle on August 11, the third cycle on September 3, and the fourth cycle on September 24. Over these four cycles, the lesions showed significant shrinkage. However, after the fifth cycle on October 16, 2023, the intraoral lesion suddenly enlarged, prompting a MDT recommendation for additional local surgery, which was performed on November 8, 2023. Twenty days post-surgery, on November 28, 2023, Pembro maintenance therapy commenced. Follow-up continued with no recurrence or metastasis observed. AR, adverse reaction; Chemo, chemotherapy; Pembro, pembrolizumab; Nimo, nimotuzumab; PD, progressive disease; PR, partial response; MDT, multidisciplinary team.

After two cycles of immune combined targeted therapy, both the left submandibular and primary lesions exhibited significant reduction (**Figures 2A**, b). After the third cycle, the lesions further reduced (**Figures 2A**, c, d). By the end of four cycles, the intraoral and submandibular lesions had regressed almost completely. According to the Response Evaluation Criteria in Solid Tumors (RECIST) version 1.1, the patient achieved a partial response (PR). Nonetheless, after the fifth cycle, tumour proliferation recurred at the primary site of the left lower gingiva (**Figures 2A**, e). After immune combined targeted therapy, the patient’s general condition significantly improved, and the willingness for treatment markedly increased owing to the perceived hope of the treatment. Additionally, the patient’s Eastern Cooperative Oncology Group Performance Status (ECOG PS) score improved from 3 to 1. Based on the patient’s physical condition and disease status, MDT recommended a local surgical intervention. Subsequently, extended resection of the primary tumour site and neck lymphadenectomy were performed. Preoperative CT scans revealed a significant reduction in the lesion size (**Figure 2B**, c-d). On November 8, 2023, pathological examination following conversion surgery revealed that the tumour cells were predominantly spindle-shaped (**Figures 1B**, a–d), with significant tumour degeneration and necrosis in certain areas (**Figures 1B**, e), accompanied by histiocytic reaction and abundant lymphocytic infiltration (**Figures 1B**, f), indicative of post-treatment changes. No tumour metastasis was observed in the dissected cervical lymph nodes. IHC results showed: CK (focal +), Ki-67 (+, 70%), CK5/6 (-), p40 (-), p16 (-), HMB45 (-), p63 (-), S100 (-), INI-1 (+), MyoD1 (-), Vimentin (+), Brg-1 (+), p53 (++, 60%), and EGFR (+) (**Figures 3B**, a–h). Based on the histological morphology and IHC results, the pathological diagnosis was confirmed as SC. Twenty days postoperatively (November 28, 2023), the patient continued adjuvant monotherapy with pembrolizumab. A CT scan five months post-surgery showed good recovery at the original lesion site (**Figure 2B**, e-f), and the patient has been followed up for one year without recurrence. No adverse reactions were observed during the treatment. The comprehensive timeline of the patient’s diagnosis, treatment, and lesion changes is depicted in **Figure 4**.

## Discussion

HNUC is a poorly differentiated and aggressive disease, with limited consensus on optimal management owing to its rarity (2, 11). Sakamoto et al. found that cetuximab-targeted therapy could produce favourable outcomes in oral UC (6). Studies indicate that over 50% of patients with UC aberrantly express the PD-L1 protein, suggesting potential sensitivity to immune checkpoint inhibitors (12). Consequently, immune checkpoint inhibitors and targeted therapies have emerged as promising therapeutic strategies for UC management. According to our clinical experience with 150 patients treated with immune-based treatment regimens, we found that some patients achieved positive outcomes after 2-4 cycles of treatment (13). However, immune resistance and disease progression may be observed in cycles 4-6, highlighting the need for incorporating local therapy. Consistent with this trend, our patient experienced significant tumour shrinkage and partial response after 4 cycles of combined immune-targeted therapy. Nonetheless, the emergence of a new tumour at the primary site following cycle 5 was indicative of disease progression.

Currently, determining the precise timing and optimal approach for incorporating local therapies after immunotherapy or targeted therapy remains a challenge. In our case, immunotherapy and targeted therapies provide new hope by converting initially inoperable tumours into surgically manageable lesions through a process known as ‘conversion therapy’ (14). Additionally, we considered the risks associated with radiation therapy, such as osteonecrosis and skin infections, and the potential increase in surgical complexity and complications if radiotherapy were to fails (15, 16). Finally, the MDT recommended surgery as a local treatment strategy. The findings of this unique case provide empirical evidence supporting the use of conversion therapy for HNUC.

Histological transformation can be considered a form of acquired resistance, and may be correlated with phenotypic alterations in the tumour induced by immunotherapy or targeted therapy (7, 17–19). In the context of lung cancer treatment, lung adenocarcinoma has been observed to transform into SCLC or more aggressive SC (7, 20–23). Liang et al. suggested a potential association between the transformation of lung adenocarcinoma to SC and the use of immune checkpoint inhibitors (7). Additionally, existing literature indicates that the transformation of lung adenocarcinomas into SC may also be linked to EGFR-targeted therapy (8). These histological changes may contribute to therapeutic resistance and a poor prognosis (10). A clear transition from UC to SC is crucial for guiding treatment strategies. A notable aspect of this case is the significant difference in the tumour’s histological morphology before and after surgery. Preoperatively, the tumour exhibited an epithelial-like cell morphology, whereas postoperatively, it showed spindle cell morphology, with no evidence of squamous or glandular differentiation (24, 25). Comprehensive IHC testing was performed both preoperatively and postoperatively, ruling out other rare malignancies such as melanoma (HMB45 and S100 negative), lymphoma (LCA negative), angiosarcoma (ERG negative), and rhabdomyosarcoma (MyoD1 negative) (24–26). The preoperative diagnosis of UC was confirmed, and the postoperative loss of INI-1 or BRG-1 further supported the diagnosis of SC. In our case, the patient successfully underwent conversion therapy before surgery, and postoperative pathology revealed a transition from UC to SC.

Although SC is predominantly found in the lungs and kidneys, it is uncommon in the head and neck region and accounts for approximately 1% of all HNC cases (2, 27, 28). SC may require a distinct therapeutic approach compared to UC. The histological transformation observed in this case suggests that repeated pathological examinations may be necessary for disease progression to potentially guide adjustments in therapeutic strategies for improved outcomes. Additionally, we observed that SC transformation induced by immune-targeted therapies may differ from that induced by radiotherapy. Based on relevant reports and our case, we found that the latency of radiotherapy-induced SC transformation may be over a period of years, whereas immune-targeted therapies can induce more rapid transitions (7, 29). This finding has potential implications for clinical work. Further investigation is needed to understand the mechanisms and clinical implications of this phenomenon, including its predictive value for prognosis and its impact on treatment strategies. Interestingly, preoperative imaging revealed enlarged cervical lymph nodes with enhancements suggestive of metastasis. However, postoperative pathology revealed thickened lymph node capsules without evidence of carcinoma. This discrepancy between imaging and pathology may indicate fibrotic repair following the regression of metastatic cancer cells in the lymph nodes owing to immunotherapy. In such cases, a fine-needle aspiration biopsy of the lymph nodes is recommended to minimise patient trauma.

This study highlights the importance of molecular matched therapies for rare malignancies without a standard of care. The combination of a PD-1 inhibitor with an EGFR inhibitor was effective and well tolerated by the patient, making conversion surgery feasible. Additionally, monitoring pathological transformations related to combined immune and targeted therapies is essential. Once disease progression occurs, rebiopsy can aid in diagnosing and managing the disease and providing timely guidance for adjusting the treatment plan.

## Data Availability

The original contributions presented in the study are included in the article/supplementary material. Further inquiries can be directed to the corresponding authors.
